# Death of Professor Richardson, of Transylvania University

**Published:** 1845-10

**Authors:** 


DEATH OF PROFESSOR RICHARDSON, OF TRANSYLVANIA UNIVERSITY.

By certain publications contained in the Lexington Observer of the 20th instant, we are informed of the death of Professor Richardson, of that city. On what day it occurred, and whether it was sudden or otherwise, is not mentioned. The following are the proceedings of his colleagues in reference to the event.

At a meeting of the Medical Faculty of Transylvania University, convened on the 15th instant, in consequence of the decease of the Professor of Obstetrics and the Diseases of Women and Children, the following minute was adopted:

Whereas, It has pleased Almighty God, in his infinite wisdom, to remove from our midst, our late colleague, Professor Richardson, who for a quarter of a century has been identified with the interests and well-being of the Medical School, therefore,

*Resolved*, That we deeply lament the loss of a colleague with whom we have been so long and so intimately associated, and whose influence has contributed, in no small degree, to advance the interests of Transylvania, and the profession in the West.

*Resolved*, That we will attend the funeral of the deceased, and so pay the last tribute of respect to his mortal remains; and that a copy of this minute be sent to the family of the deceased, and also to the Observer and Reporter, for publication.

By order,

THOMAS D. MITCHELL, M. D., Dean of Faculty.

*Lexington*, Sept. 15th, 1845.

Professor Mitchell is to discharge the duties of the chair of Obstetrics, in addition to those of Materia Medica, during the coming session—in the mean time the Trustees of the University have issued a notice, inviting applications for the vacant Professorship, to be forwarded to the Dean of the Faculty, prior to the 30th day of January next.

